# Comparison of standard, quantitative and digital PCR in the detection of enterotoxigenic *Bacteroides fragilis*

**DOI:** 10.1038/srep34554

**Published:** 2016-09-30

**Authors:** Rachel V. Purcell, John Pearson, Frank A. Frizelle, Jacqueline I. Keenan

**Affiliations:** 1 Department of Surgery, University of Otago, 2 Riccarton Avenue, PO Box 4345, Christchurch 8140, New Zealand; 2 Department of Population Health,University of Otago, 2 Riccarton Avenue,PO Box 4345,Christchurch 8140, New Zealand

## Abstract

Gut colonization with enterotoxigenic *Bacteroides fragilis* (ETBF) appears to be associated with the development of colorectal cancer. However, differences in carriage rates are seen with various testing methods and sampling sites. We compared standard PCR, SYBR green and TaqMan quantitative PCR (qPCR) and digital PCR (dPCR) in detecting the *B. fragilis* toxin (*bft*) gene from cultured ETBF, and from matched luminal and faecal stool samples from 19 colorectal cancer patients. Bland-Altman analysis found that all three quantitative methods performed comparably in detecting *bft* from purified bacterial DNA, with the same limits of detection (<1 copy/μl). However, SYBR qPCR under-performed compared to TaqMan qPCR and dPCR in detecting *bft* in clinical stool samples; 13/38 samples were reported positive by SYBR, compared to 35 and 36 samples by TaqMan and dPCR, respectively. TaqMan qPCR and dPCR gave *bft* copy numbers that were 48-fold and 75-fold higher for the same samples than SYBR qPCR, respectively (*p* < 0.001). For samples that were *bft*-positive in both fecal and luminal stools, there was no difference in relative abundance between the sites, by any method tested. From our findings, we recommend the use of TaqMan qPCR as the preferred method to detect ETBF from clinical stool samples.

The association between gut colonization with particular bacterial species, and the development of colorectal cancer, has been reported by our group and several others^1^,^2^,^3^. Prominent among these bacterial species is enterotoxigenic *Bacteroides fragilis* (ETBF). *B. fragilis* is a gram negative commensal bacteria and colonic carriage has been reported in up to 80% of people. Colonization with ETBF that carry a gene coding for a metalloprotease, *B. fragilis* toxin (BFT) 1, 2 or 3, has been reported in up to 30% of the population^4^.

*In vitro* studies have shown that ETBF results in the cleavage of E-cadherin^5^,^6^, resulting in β-catenin signalling and colonic epithelial cell proliferation^7^ that promotes epithelial-mesenchymal transition, a process involved in carcinogenesis. ETBF has also been shown to promote chronic inflammation in the gut by stimulating secretion of the pro-inflammatory cytokine, IL-8^9^, and by Stat3 activation^10^, which may also drive tumorigenesis. Although the exact mechanisms by which ETBF contributes to the development of colorectal carcinogenesis have yet to be elucidated, the presence of these bacteria may represent an early indicator/risk factor of colorectal neoplasia.

The presence of ETBF has been reported in the stool samples of colorectal cancer patients in several studies^1^,^3^,^11^, and a sensitive method of faecal detection of these bacteria may be important in future colorectal screening programmes. However, bacterial detection in faecal stool samples may not be a true reflection of the microbial population at different locations in the gut. The majority of reports published to date regarding the gut microbiome have looked exclusively at faecal stool samples. A recent study by Li *et al*., has demonstrated a higher level of microbial diversity in luminal stool and tissue samples from the duodenum, compared to samples taken from the rectum, and to faecal samples^12^. Detection of low abundance bacterial species may also be hindered by the dilutive effect of faecal matter and other more abundant bacteria, and the inhibitory effects of bile salts, and faecal proteins and enzymes on the detection methods used^13^.

The use of standard (end-point) PCR has been described in several studies to detect ETBF in faecal samples, and a recent study by Sears’ group has used touch-down PCR and quantitative PCR (qPCR) to detect faecal ETBF with greater sensitivity^14^. Absolute quantitation using qPCR requires the use of a standard dilution series, and the accuracy of qPCR decreases with lower target abundance, due to the logarithmic nature of PCR. Digital PCR (dPCR) is a third generation PCR platform that allows direct absolute quantification without the need for standard curve generation^15^, and it has also been shown to tolerate PCR inhibitors better than qPCR^16^. The aim of this study was to compare the different methods previously described (standard PCR, SYBR green and TaqMan quantitative PCR), in addition to digital PCR, in detecting the *B. fragilis* toxin (*bft)* gene from cultured ETBF DNA, and from matched luminal and faecal stool samples.

## Methods

We analysed a dilution series of DNA extracted from purified ETBF in addition to matched pairs of pre-operative faecal samples and luminal stool samples, adjacent to the tumour site, from 19 patients with diagnosed colorectal cancer, and compared the performance of standard PCR, qPCR, using both SYBR green and TaqMan technology, and dPCR in detecting ETBF.

### Patients

Nineteen patients with diagnosed colorectal adenocarcinoma provided pre-operative faecal stool samples. Patients did not undergo bowel preparation prior to surgery, allowing matched luminal stool samples, adjacent to the tumour site, to be taken at the time of surgery. None of the patients had received preoperative chemotherapy or radiation. This study was approved by the Human Ethics Committee (Health) of the University of Otago, and was carried out in accordance with its guidelines. All patients provided written informed consent.

### Reference strains

Three ETBF strains containing the three Bft subtypes were generously supplied by Professor Cynthia Sears, Baltimore, USA, and were used as reference strains in this study: VPI 13784 (Bft-1)^17^, 86-5443-2-2 (Bft-2)^18^, and Korea 570 (Bft-3)^19^. *Fusobacterium nucleatum* (Fn) and *E. coli* were used as specificity controls. The ETBF and Fn strains were cultured anaerobically, and *E. coli* was cultured aerobically, all on sheep blood agar (Fort Richard Laboratories, Auckland, New Zealand).

### DNA extraction

DNA was extracted from colonies of reference strains using DNeasy Blood and Tissue Mini Kit (Qiagen, Hilden, Germany), as per the manufacturer’s instructions for gram-negative bacteria. DNA extraction included digestion with Proteinase K for 3 hours at 56 °C. DNA was extracted from approximately 200 mg stool samples using QIAmp DNA Stool Mini Kit (Qiagen), as per the manufacturer’s instructions. Purified DNA was quantified using the NanoDrop 2000c spectrophotometer (Thermo Scientific, Asheville, NC, USA). DNA samples were stored at −20 °C.

### PCR primers

Two different sets of primers were used in this study; primers for standard PCR and qPCR using SYBR-green were taken from a study by Odamaki *et al*.^20^, while the primer/probe set used in TaqMan qPCR and dPCR was that published by Chen *et al*.^14^. Both sets of primers were tested in silico (BLAST search) and showed 100% identity to all three published BFT isotypes, and were also successfully used to amplify *bft* from each of the three BFT isotypes. Amplicons were purified and sequenced from standard and SYBR qPCR to verify primer specificity. Primer sequences are shown in Table 1.

### Standard PCR

Each PCR reaction contained 500 nM of each primer, 200 nM dNTPs, 2 mM MgCl_2_, 1X enzyme buffer, 0.5 U HotFire Polymerase (Solis Biodyne, Tartu, Estonia), and 25–35 ng DNA template (1 μl). in a 10 μl reaction volume. Reactions were carried out on a SuperCycler thermal cycler (Kyratec, Mansfield, Queensland, Australia), and thermal cycling conditions were as follows: 15 mins at 95 °C, followed by 35 cycles of 30 sec at 95 °C, 30 sec at 66 °C and 30 sec at 72 °C, and a final extension step of 2 min at 72 °C. Products were visualised on a 1.5% agarose gel containing SYBRSafe (Invitrogen, Carlsbad, CA, USA). A no template control (NTC), and DNA extracted from *Fn* and E. *coli* (specificity control) were included in each PCR run.

### Quantitative PCR

Quantitative polymerase chain reaction (qPCR) was carried out to quantify levels of ETBF in stool samples using the LightCycler^®^480 thermocycler (Roche Diagnostics, Indianapolis, IN, USA). For reactions using SYBR-green chemistry, each 10 μl reaction consisted of 25–35 ng of genomic DNA (1 μl), 500 nM of each primer (see Table 1), 5 μl of SYBR Green Master Mix (Roche Diagnostics), and 1.5 μl of water. Thermal cycling conditions were as follows: 1 cycle of 95 °C for 5 mins, followed by 50 cycles of 95 °C for 10 secs, 65 °C for 10 secs and 72 °C for 20 secs. Melting curves were obtained by heating samples from 65 °C to 97 °C in increments of 0.11 °C/s with 5 acquisitions per °C.

Quantitative PCR reactions using TaqMan consisted of 5 μl TaqMan Fast Advanced Master Mix (Applied Biosystems), 0.5 μl TaqMan primer/probe (final concentrations of 900 nM/250 nM, respectively; see Table 1 for probe and primer sequences) and 25–35 ng of genomic DNA (1 μl) in a 10 μl reaction. Thermal cycling conditions were as follows: 1 cycle of 95 °C for 10 mins, followed by 50 cycles of 95 °C for 10 secs and 60 °C for 20 secs.

The concentration of *bft* was expressed as genome copy numbers by calculating the weight of one ETBF genome, as described by Dolezel *et al*.^21^. ETBF has an estimated genome size of 5.2 Mb, and a single ETBF genome weighs approximately 5.31 fg (5.2 Mb/978Mb [one picogram of double stranded DNA] = 0.00531 pg). Therefore, 1 ng of ETBF DNA contains approximately 188,680 copies of the genome (1000 pg/0.00531 pg). DNA extracted from purified ETBF (strain VPI13784) had a measured concentration of 170 ng/μl (32075600 copies of the ETBF genome), and standard curves were made by serially diluting this DNA to establish limits of detection (LOD), and for use in absolute quantitation of DNA from stool samples.

Quantitative PCR raw data analysis was performed using the Absolute quantification/2^nd^ derivation max module of the LightCycler 480 software (Roche). PCR efficiencies, calculated from standard curves, were >98% for both SYBR and TaqMan qPCR. All qPCR reactions were performed in triplicate. NTCs and specificity controls were included in each qPCR run.

### Digital PCR

The QuantStudio 3D Digital PCR system (Life Technologies) was used to carry out dPCR. Samples, consisting of 9 μl QuantStudio 3D Mastermix, 1.8 μl of genomic DNA (25–35 ng/μl DNA), 0.9 μl of TaqMan primer/probe set (final concentrations of 900 nM/250 nM, respectively), in a final volume of 18 μl, were loaded onto chips using the QuantStudio 3D Digital Chip Loader. The chips were sealed and thermal cycling was carried out on a GeneAmp^®^ PCR system 9700 thermocycler (Applied Biosystems), adapted for dPCR chips, using the following parameters: 96 °C for 10 mins, followed by 50 cycles of 60 °C for 2 mins and 98 °C for 30 sec, followed by a final extension at 60 °C for 2 mins. The chips were then loaded onto the QuantStudio™ 3D Digital PCR Instrument, and the end-point fluorescence of each partition on the chips was measured using QuantStudio™ 3D Digital PCR software v3.0. Each sample was carried out in duplicate and an NTC was included with each run. Digital PCR was also carried out on DNA extracted from *Fn* and *E coli*, using the primer/probe set for *bft*, as specificity controls.

**Figure 1:**
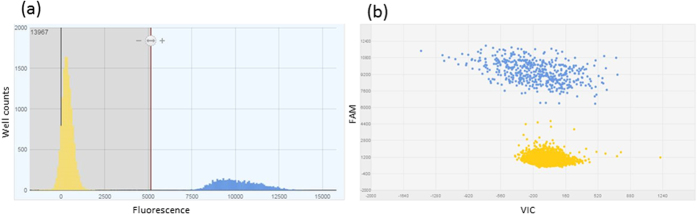
An example of a digital PCR histogram and scatterplot generated using QuantStudio 3D AnalysisSuite Cloud Software. (**a**) Representative histogram of *bft* gene detected using dPCR from a clinical stool sample (in blue); the yellow peak represents wells on the dPCR chip containing no target. The histogram is generated by plotting fluorescence versus well count for every well on the dPCR chip. The red vertical line represents the call threshold calculated by the software. This can be manually user defined to give an acceptable balance of quality and quantity data. (**b**) The corresponding scatter plot, showing each of the wells with target amplification (blue) or no target (yellow) as a discreet dot. There is acceptable definition between amplified and negative wells; this corresponds to the definition between histogram peaks.

Raw data analysis was performed using QuantStudio 3D Analysis Suite Cloud Software v3.0.2.2 for dPCR. Each chip was manually reviewed and the threshold was user defined to achieve an acceptable balance of quality and quantity data (Fig. 1). As the QuantStudio software calculates an absolute concentration of the target per μl of input DNA, it allowed us to compare quantitation across methods with the same input DNA concentration (25–35 ng DNA) for each sample.

### Statistical analyses

Linear models of standard deviations on mean values showed evidence that measurement error increased with mean value for methods in both clinical samples and standards (all *P* < 0.00001), hence log_10_ concentrations were analysed to assess precision and method comparability. Precision was quantified using the within-sample coefficient of variation (CV), a dimensionless percentage, while methods were compared using Bland-Altman plots and limits of agreement (LOA) at the 95% level of confidence calculated^22^,^23^. Bland-Altman analysis uses mean values to report the average ratio between methods, effectively a measure of bias; the 95% LOA predict a range that cover 95% of ratios. A mean ratio of 1 shows that no bias exists, while a ratio of 10 shows that one method gives results that are, on average, 10 times the other method. An example of a Bland-Altman analysis with LOA of [0.9, 1.2] means that 95% of the measurements for method 2 are expected to be between 10% lower and 20% higher than method 1, which we consider to be relatively good agreement for PCR methods. LOA of [0.01, 100] imply that 95% of the results generated by the two methods being compared differ by 2 orders of magnitude, which we consider to be poor agreement for PCR methods.

Comparison of luminal and fecal samples was carried out via a t-test, with Sattherwaites adjustment for unequal variances on the log-transformed concentrations for subjects with both luminal and fecal positive samples. Digital PCR had a zero value for two samples so the dPCR data was transformed with log concentration plus 4 (the 5% quantile of dPCR concentrations). All measurements have been back-transformed to the natural scale. Analysis was performed in the R language for statistical computing version 3.2.4 (Vienna, Austria) and all statistical tests are two sided, with a type I error rate of 5%.

## Results

### Specificity analysis

All four methods were highly specific and showed 100% concordance (k = 1.0). No *E. coli or F. nucleatum* samples were detected by any method and all methods correctly identified positive controls. No false positives were detected using NTCs, by any method. Amplified products from standard and SYBR qPCR were sequenced using Sanger sequencing, and the resulting sequences were searched against online DNA databases using BLAST search, in order to verify primer specificity. All amplicon sequences were found to be either *bft*-1 or *bft*-2.

### Limits of detection of PCR methods

End-point analysis using standard PCR of serially diluted purified ETBF showed a limit of detection of approximately 32 copies of the genome/1 μl input DNA after 35 cycles of amplification. Samples were analyzed in triplicate using both qPCR methods, and a detectable signal was observed for each replicate in the dilution series containing ~0.32 copies of the genome/μl. No amplification was seen in any replicate containing 0.032 genome copies/μl. Dilution series samples were analysed in duplicate using the specific TaqMan primer/probe set by dPCR. No measurement was obtainable from the highest dilution (~320756 copies/μl), due to saturation of the digital chip with positive signal. No detectable signal was observed from the lowest dilution (0.032 genome copies/μl), giving a detection limit of ~0.32 copies/μl.

### BFT detection in clinical stool samples

**Figure 2:**
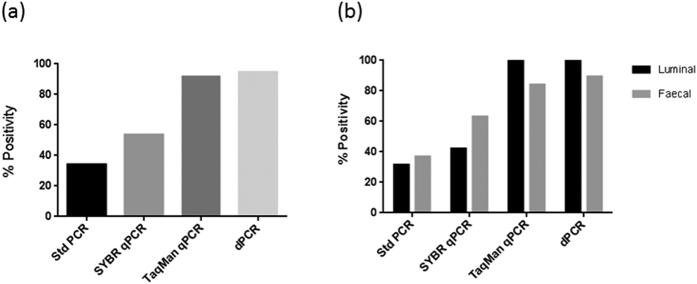
The overall percentage of *bft*-positive samples as determined by each of the PCR methods is shown in (**a**), while (**b**) shows the percentage positivity of luminal versus faecal stool samples using the different PCR methods.

*Bft*-positivity, as measured by each PCR method, is given in Supplementary Table 1, and Fig. 2 shows the overall *bft*-positivity, and percentage positivity for luminal and faecal stool samples as determined by each of the PCR methods. *Bft* was detected in 13/38 stool samples from 10/19 patients, using standard PCR. These comprised three matched pairs of luminal and faecal samples, and an additional three luminal and four faecal samples. *Bft* was detected in an additional ten samples using SYBR green qPCR. These comprised three luminal and seven faecal samples. However, three of these samples were only detected in a single replicate close to the threshold cut-off of 40 cycles, and were not included in subsequent analysis. Hence, 23/38 samples from 13/19 patients were positive using SYBR green qPCR. Quantitative PCR using TaqMan chemistry detected the presence of *bft* in 35/38 stool samples from 19/19 patients; three faecal samples were negative for *bft*. *Bft* was detected in all but two of the faecal samples using dPCR, a total of 36/38 samples from 19/19 patients. Table 2 shows the pairwise agreement of *bft* status of each sample as determined by each PCR method. The strongest agreement was found between TaqMan qPCR and dPCR. Only samples positive in two or more replicates by qPCR and in both replicates by dPCR were used for subsequent analysis. There was no difference detected in mean *bft* copy number between fecal and luminal samples with any method tested (all *P* > 0.7) (Supplementary Table S2).

### Comparison of nominal input values and calculated BFT concentrations

Bland-Altman analysis was carried out to compare nominal input concentration (based on spectrophotometric calculations) and absolute concentration calculated by the three quantitative PCR. Both qPCR methods show high agreement with the input concentrations with mean ratios (95% LOA) of 1.02 [0.90, 1.15] and 0.996 [0.85, 1.17] for SYBR and TaqMan, respectively. The least agreement was seen between dPCR results and the nominal input concentrations, with a mean value of 1.21 [0.32, 4.64]. However, this disagreement was mainly due to the lowest concentration in the dilution series (<1 copy/μl), and when this was removed from the analysis, the agreement between dPCR values and nominal input values improved to a ratio of 1.03 [0.70, 1.51].

### Comparison of quantitative PCR methods in the detection of *bft* in the dilution series

Measurement of the geometric error shows that SYBR qPCR was the most precise, with a geometric standard deviation of 1.08 (CV = 0.08). However, all three methods had acceptable levels of precision over the range of concentrations tested (Table 3).

**Figure 3:**
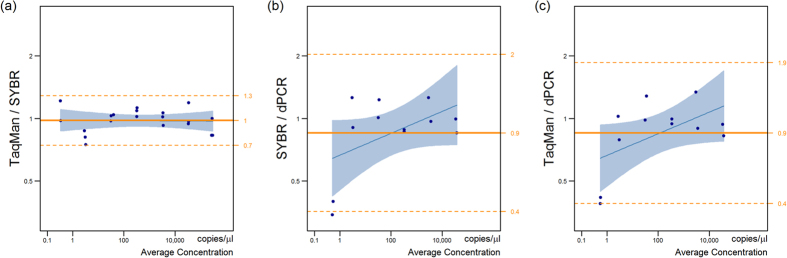
Bland-Altman analysis on log-transformed results of the quantification of *Bft* from a dilution series of purified ETBF DNA was used to analyse the agreement between. (**a**) SYBR and TaqMan qPCR, (**b**) SYBR qPCR and dPCR and (**c**) TaqMan qPCR and dPCR. 95% limits of agreement are given on the right-hand axis.

Average ratios and limits of agreement (Table 4), for pairwise comparisons of the three quantitative methods on the dilution series data, can be used to summarise the Bland-Altman plots (Fig. 3). There was no evidence of systematic bias between any methods (all P > 0.14), although dPCR did produce measurements on average 15% lower than SYBR and Taqman. The limits of agreement for the pairwise comparisons show acceptable agreement. The least agreement is seen between Taqman and dPCR, which predicts that 95% of measurements would be between 3.2 times lower or 2.45 times higher using dPCR; however, this is well within one order of magnitude.

This comparative analysis of the three quantitative methods for measuring *bft* showed that there were no significant differences between the methods when used to detect *bft* in purified DNA samples over a specified concentration range.

### Comparison of methods in detecting *bft* in clinical stool samples

Geometric measurement of error in clinical samples shows that SYBR green qPCR performs the least well of the three methods and TaqMan qPCR performs the best (geometric measurement error 3.42 and 1.25, respectively; CV = 2.42 and 0.25, respectively; Table 3).

**Figure 4:**
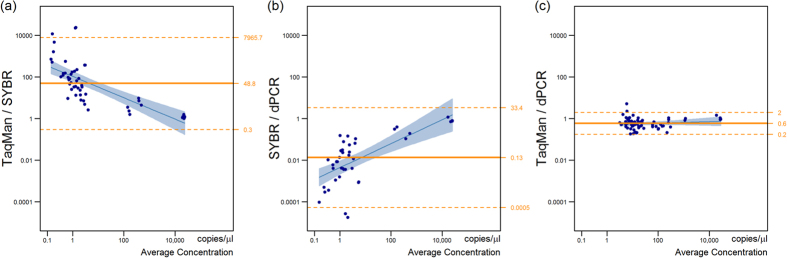
Bland-Altman analysis on log-transformed results was used to analyse the agreement between (**a**) SYBR and TaqMan qPCR, (**b**) SYBR qPCR and dPCR and (**c**) TaqMan qPCR and dPCR), in the quantification of *Bft* in a series of clinical stool samples. 95% limits of agreement are given on the right-hand axis.

Bland-Altman analysis (Fig. 4) shows significant bias between all three methods (all *P* < 0.001). TaqMan qPCR and dPCR are the most comparable techniques for measuring *bft* in clinical stool samples, with a mean ratio of 0.6 and 95% LOA of [0.18, 1.82]. This means that 95% of measurements by TaqMan are expected to be between 5.6 times lower or 1.8 times higher than dPCR, well within an order of magnitude.

SYBR qPCR produces measurements on average 48 times lower than Taqman qPCR, and 75 times lower than dPCR (95% LOA of [0.3, 8000] and [0.3, 19000], respectively). The ratios have been inverted to put SYBR qPCR in the denominator. Bland Altman plots (Fig. 4a,b) show that this difference is particularly high at low concentrations.

These results, together with the number of *bft*-positive samples detected by each method (Supplementary Table 1), show that SYBR green qPCR appears to be least reliable method for detecting BFT in clinical stool samples and least comparable to the other methods. TaqMan qPCR and dPCR perform comparably well in a clinical setting, with average agreement well within an order of magnitude.

### Cost analysis

A comparative analysis of the cost of each PCR method was carried out including reagents and consumables and showed that standard PCR costs approximately US$0.20 per replicate. Out of the quantitative methods, SYBR qPCR was the most cost effective at US$1/replicate, TaqMan qPCR cost US$1.75/replicate and dPCR cost almost five times that amount at US$8.50/replicate. Up to 384 replicates could be analysed in less than 3 hours, including sample preparation using both qPCR methods, while a maximum of 24 samples could be analysed at a time using dPCR, and this took up to 5 hours.

## Discussion

*B. fragilis* is a commensal bacteria commonly found in the gut. Enterotoxigenic strains of *B. fragilis* that contain the gene encoding BFT have been linked to the development of colorectal cancer (CRC) in humans and mice. The reported of rate carriage of ETBF in CRC varies widely, and may be attributable to different methodologies used, in addition to geographical variation in carriage rates of the bacteria^9^. We aimed to compare the some of the more commonly reported methods used to detect ETBF, in addition to the more recently described digital PCR, in order to establish a “gold standard’ method. The most commonly described technique to detect the presence of the *bft* gene in faecal stool samples has been standard PCR, and a study by Toprak *et al*. reported carriage rates of 38% in CRC patients^1^. Here, we detected ETBF in faecal and/or luminal stool samples from 10 of 19 CRC patients (53%), using standard PCR. This higher rate of detection may be due to the small sample size in our study. We have also previously identified that the choice of PCR primers to detect ETBF greatly affects the specificity of PCR, and this may account for the lower detection rate in the study by Toprak’s group^24^. A study by Boleij *et al*. found that ETBF was present at a much higher rate of 85.5% in mucosal tissue samples of CRC patients, using touchdown standard PCR^2^. Standard PCR is very sensitive to inhibitors, such as bile salts, complex polysaccharides and urates found in stool samples^13^. Therefore, although we could detect ETBF to 32 copies/μl using this method, this was from purified bacterial DNA, and similar numbers of ETBF may not be detectable in clinical stool samples due to the action of inhibitors. Differing sample types used (tissue versus stool) may also contribute to the discrepancy between our study findings.

A further study by Viljoen *et al*. examined carriage of ETBF in colonic mucosal tissue samples using SYBR green qPCR^11^. Their findings of ETBF in 26% of CRC patients is considerable lower than that reported by Boleij *et al*. using the same sample type, and by our group using the same technology on stool samples in the current study (74%). The choice of primers used in the study by Viljoen’s group may explain these discrepancies; the primers used in that study were designed against a DNA sequence predicted from the amino acid sequence of the BFT toxin, and do not show complete identity to the since published *bft* genes. In particular, they contain mismatches to *bft*-2 and *bft*-3, which may lead to false negative results^24^. In contrast, the primers used in this study^14^,^20^ show complete identity to published sequences of all three BFT isotypes.

Although a published study by Chen *et al*. used TaqMan probes to detect ETBF^14^, our study is the first to apply this method to examine the carriage of ETBF in CRC patients, and the first to quantitate ETBF from clinical stool samples. Our study is also the first to examine the use of digital PCR as a means of detecting ETBF. Both TaqMan qPCR and dPCR, which make use of the same primer/probe set, detected ETBF in the majority of clinical samples in our study: 35/38 and 36/38 samples, respectively.

We found no difference in the relative abundance of ETBF between luminal and fecal stool samples where both were positive for the presence of the bacteria. This implies that testing for the presence of ETBF in fecal stool samples provide an accurate reflection of the presence of ETBF in luminal stool samples adjacent to the tumour site, and this may have implications for screening. It should be noted that investigation of the presence of ETBF in the mucosa adjacent to the tumour or in the tumour tissue itself was beyond the scope of this study, and therefore no inference can be made regarding ETBF at these sites; analysis of the association of tumour-associated ETBF with its abundance in fecal stool samples represents an important investigation for future studies.

The abundance of bacteria detected ranged from 0.28–22319/μl, 3.34–24969/μl and 4.4-29096/μl by SYBR green qPCR, TaqMan qPCR and dPCR, respectively. The numbers of bacteria detected in stool samples by both qPCR methods were consistently lower than those detected for the same samples using dPCR. This is likely due to the fact that qPCR uses a standard curve generated using serially diluted DNA from purified ETBF, and the number of genome copies in the stock sample was calculated based on spectrophotometric determination of the DNA concentration. On the other hand, quantification using dPCR is independent of a standard curve. Instead, the sample is divided into a large number of partitions, and the end-point fluorescence of each partition is measured, and Poisson-statistics are used to measure the absolute number of targets in the sample^15^,^16^. Therefore, quantification using dPCR may be more accurate than calculations from qPCR experiments.

The three quantitative methods detected *bft* from purified ETBF DNA to the same lower limits, showing similar sensitivity, despite the use of two different primer sets; that for SYBR qPCR and the TaqMan primer/probe set. None of the methods detected amplification in the no template controls or specificity controls, implying equal specificity. All methods had acceptable precision (<10%) to a limit of 320 copies/μl input DNA. As expected, precision of the methods decreased with decreasing concentrations, with dPCR being the least precise at the lower detection limits. To compare the agreement between the methods, we used Bland-Altman analysis as it accounts for both bias and reproducibility and works well on the log scale. Bland-Altman plots showed that there was no significant differences between the methods when used to detect *bft* in purified DNA samples, i.e. the methods showed high levels of agreement.

However, when used to detect ETBF in clinical stool samples, SYBR green qPCR only detected ETBF in 20 samples, compared to 35 and 36 samples using TaqMan qPCR and dPCR, respectively. Quantitative measurements produced by SYBR qPCR were, on average, 48 times lower than Taqman qPCR, and 75 times lower than dPCR. Taken together, these results suggest that SYBR qPCR may be affected by PCR inhibitors, present in clinical stool samples. This reflects previous findings that TaqMan qPCR is less affected by PCR inhibitors than SYBR chemistry, and that dPCR performs better than qPCR in the presence of inhibitors, and on clinical stool samples^16^. However, as a different primers were used for SYBR green qPCR, it is not possible to definitively rule out inhibition of the primer set used, rather than inhibition of the SYBR green chemistry itself, by inhibitors in the stool samples. Bland-Altman analysis of qPCR and dPCR using the same TaqMan probe set and the same annealing temperature gave comparable results, with dPCR detecting one additional positive clinical sample.

Although this study has detected a much higher carriage rate of ETBF than previously described in stool samples^1^,^3^, our results are comparable to the higher rates of detection (88.5%) seen in mucosal tissue of CRC patients using touchdown PCR^2^. The low number of cases was a limitation of our study, and a larger study with greater patient numbers should be carried out to verify our findings regarding the carriage rates of ETBF in CRC. Our use of Bland-Altman analysis allowed us to measure the agreement between methods, and detect a constant bias in a particular method, if present. This analysis does not provide any information on the effects of individual components of a method, such as primers or thermocycling conditions that may contribute to its performance, although our use of DNA from clinical samples in addition to purified bacterial DNA, allows us to infer about the possible source possible bias.

This study highlights the importance of method selection when investigating the presence of bacteria in stool samples. This is particularly important in the case of pathogenic bacteria that may play a role in the development of cancer. Low levels of bacteria may not be detected by conventional methods, and screening methods for pathogenic bacteria for use as biomarkers or disease indicators need to be robust and sensitive. Although all three quantitative methods performed well in detecting *bft* in purified DNA, SYBR green qPCR performed less well in the detection of *bft* in clinical stool samples, highlighting the need to test “real” samples prior to implementation of a new detection method.

In this study, we found that quantitative PCR using TaqMan was comparable to digital PCR in detecting low abundance targets from clinical samples, and the lower costs and increased number of samples that can be processed in a single run makes it more applicable for use in a diagnostic setting. However, the use of digital PCR is recommended when more accurate pathogen counts are needed, e.g. when cut-off values have been established that discriminate between commensal colonization and disease causation. The use of digital PCR is also recommended to establish the concentration of a calibration or reference target for subsequent use in other platforms, such as qPCR.

## Additional Information

**How to cite this article**: Purcell, R. V. *et al*. Comparison of standard, quantitative and digital PCR in the detection of enterotoxigenic *Bacteroides fragilis.* *Sci. Rep.* **6**, 34554; doi: 10.1038/srep34554 (2016).

## Supplementary Material

Supplementary Information

## Figures and Tables

**Table 1:** Primers and probe sets used for PCR.

PCR platform	Primer sequences	Annealing temperature	Reference
Standard PCR and qPCR (SYBR green)	F: GGATACATCAGCTGGGTTGTAG	66 °C	Odamaki^20^
	R: GCGAACTCGGTTTATGCAGT		
qPCR (TaqMan) and dPCR	F: GGATAAGCGTACTAAAATACAGCTGGAT	60 °C	Chen^14^
	R: CTGCGAACTCATCTCCCAGTATAAA		
	Probe: CAGACGGACATTCTC*		

PCR, polymerase chain reaction; q, quantitative; d, digital; F, forward; R, reverse; *FAM™ dye-labelled probe with MGB quencher.

**Table 2:** Pairwise agreement of ETBF status for four different PCR methods.

		Positive Percentage Agreement		Negative Percentage Agreement		Kappa	
	(*ref*)	*PPA*	*95% CI*	*NPA*	*95% CI*	*κ*	*P*
TaqMan qPCR	dPCR	100.0%	(0.16,1.00)	97.2%	(0.85,1.00)	0.79	0.000
SYBR qPCR	dPCR	100.0%	(0.16,1.00)	47.2%	(0.30,0.65)	−0.18	0.255
Standard PCR	dPCR	100.0%	(0.16,1.00)	36.1%	(0.21,0.54)	−0.32	<10^−5^
SYBR qPCR	TaqMan qPCR	100.0%	(0.29,1.00)	48.6%	(0.31,0.66)	−0.10	0.553
Standard PCR	TaqMan qPCR	100.0%	(0.29,1.00)	37.1%	(0.21,0.55)	−0.24	<10^−5^
Standard PCR	SYBR qPCR	100.0%	(0.84,1.00)	76.5%	(0.50,0.93)	0.78	0.000

qPCR, quantitative PCR; dPCR, digital PCR; PPA, positive percentage agreement; NPA, negative percentage agreement; ref, reference method with most positive calls.

**Table 3:** Measurement of error in PCR methods in clinical and dilution series samples.

	Dilution series data		Clinical data	
	GME	CV	GME	CV
SYBR qPCR	1.08	0.08	3.42	2.42
TaqMan qPCR	1.73	0.72	1.25	0.25
dPCR	1.14	0.14	1.53	0.53

GME, geometric measurement of error; CV, within subject coefficient of variation; PCR, polymerase chain reaction, q, quantitative; d, digital.

**Table 4:** Comparison of methods using Bland-Altman analysis, showing the limits of agreement, using dilution series and clinical samples.

	Dilution series data					Clinical data				
	Mean	Lower	Upper	SD	*P*	Mean	Lower	Upper	SD	*P*
TaqMan vs SYBR	0.97	0.31	2.45	1.68	0.29	48.78	0.30	7965.74	12.78	<0.001
SYBR vs dPCR	0.85	0.36	2.03	1.54	0.39	0.01	5.35E-05	3.34	15.81	<0.001
TaqMan vs dPCR	0.84	0.39	1.83	1.48	0.14	0.59	0.18	1.94	1.82	<0.001

Lower, lower limit of agreement; upper, upper limit of agreement; SD, standard deviation; *P*, *P*-value.
