# Supplementary material for: Comparison of standard, quantitative and digital PCR in the detection of enterotoxigenic Bacteroides fragilis
Source: Sci Rep. 2016 Sep 30;6:34554. doi: 10.1038/srep34554 (PMC5043350; doi:10.1038/srep34554)
Supplement: Supplementary Information [file srep34554-s1.pdf]

Comparison of standard, quantitative and digital PCR in the detection of enterotoxigenic *Bacteroides fragilis*

Rachel V Purcell[1]*, John Pearson[2], Frank A Frizelle[1], Jacqueline I Keenan[1].

[1]Department of Surgery,
University of Otago,
2 Riccarton Avenue,
PO Box 4345,
Christchurch 8140,
New Zealand.

[2]Department of Population Health,
University of Otago,
2 Riccarton Avenue,
PO Box 4345,
Christchurch 8140,
New Zealand.

Corresponding author email: rachel.purcell@otago.ac.nz

Supplementary Table S1. Age, sex and tumour stage of patients, and ETBF status of matched faecal and luminal stool samples as determined by four different PCR methods.

Patient ID	Age (years)	Sex	Tumour stage	Stool site	Standard PCR	qPCR SYBR	qPCR TaqMan	dPCR
400	73	M	2	L	-	-	+	+
				S	+	+	+	+
401	62	F	3	L	+	+	+	+
				S	+	+	+	+
403	76	F	4	L	-	-	+	+
				S	-	-	+	+
404	86	F	3	L	-	-	+	+
				S	-	+	+	+
406	67	F	3	L	-	-	+	+
				S	-	-	+	+
408	63	M	1	L	-	-	+	+
				S	-	-	-	-
409	81	F	3	L	-	-	+	+
				S	-	-	-	+
410	74	M	3	L	-	-	+	+
				S	-	-	+	+
411	82	F	3	L	-	-	+	+
				S	+	+	+	+
412	80	M	2	L	-	-	+	+
				S	+	+	+	+
414	79	F	3	L	-	-	+	+
				S	-	-	-	-
415	78	F	2	L	+	+	+	+
				S	+	+	+	+
416	74	F	3	L	-	+	+	+
				S	-	+	+	+
417	82	M	3	L	+	+	+	+
				S	-	+	+	+
418	76	F	3	L	-	-	+	+
				S	+	+	+	+
419	84	F	4	L	-	+	+	+
				S	-	+	+	+
421	77	M	4	L	+	+	+	+
				S	-	-	+	+
422	58	M	3	L	+	+	+	+
				S	-	+	+	+
425	77	M	3	L	+	+	+	+
				S	+	+	+	+

M, male; F, female; S, faecal stool sample; L, luminal stool sample; PCR, polymerase chain reaction, q, quantitative; d, digital.

Supplementary Table S2. Comparison of luminal and fecal abundance of ETBF for matched pairs of samples. The confidence intervals and *p*-values are calculated from t-tests carried out on back-transformed log values.

Assay	n	Luminal	Fecal	95% CI	*P*-value
TaqMan	16	1514	1654	(0.2, 5.0)	0.98
SYBR	7	2654	3215	(0.0, 310.0)	0.99
dPCR	19	1166	1641	(0.3, 4.6)	0.78

n, number of ETBF-positive matched pairs; luminal and fecal abundance of ETBF is given as copies of *bft*/µl DNA
